# Multiple Chemical Sensitivity Syndrome: First Symptoms and Evolution of the Clinical Picture: Case-Control Study/Epidemiological Case-Control Study

**DOI:** 10.3390/ijerph192315891

**Published:** 2022-11-29

**Authors:** Sandra Fares-Medina, Isabel Díaz-Caro, Rebeca García-Montes, Inmaculada Corral-Liria, Soledad García-Gómez-Heras

**Affiliations:** 1 Student International Doctoral School, Rey Juan Carlos University (URJC), 28922 Madrid, Spain; 2 University Hospital Severo Ochoa, 28911 Madrid, Spain; 3 Nursing and Stomatology Department, Rey Juan Carlos University (URJC), 28922 Madrid, Spain; 4 Basic Health Science Department, Rey Juan Carlos University (URJC), 28922 Madrid, Spain

**Keywords:** multiple chemical sensitivity, idiopathic environmental intolerance, symptoms, Quick Environmental Exposure Sensitivity Inventory

## Abstract

Multiple chemical sensitivity (MCS) is a chronic condition characterized by the appearance of symptoms caused by exposure to chemical compounds that are tolerable for the general population. It mainly affects middle-aged women. There are very few studies focusing on the most frequent symptoms of MCS considering age groups and gender. The main goal of this study was to find the most frequent symptoms both at the onset of the disease and at the present time describing them by age groups. The QEESI (Quick Environmental Exposure and Sensitivity Inventory) questionnaire, Scale 3 which assesses symptoms and their severity, was used as a diagnostic tool for the disease. A case-control study was conducted with the participation of 210 people. Of the cases, 94.3% were women. The symptoms that most often manifested first were airway and mucous membrane alterations (68.9%). In the development of the disease, we found cognitive alterations (OR = 31.25), heart or chest problems (OR = 22.49), neuromuscular problems (OR = 20.00) and head-related symptomatology (OR = 19.29). Identifying the most frequent pattern of symptoms by age group and sex will allow an early diagnosis of the disease to improve its prognosis and treatment.

## 1. Introduction

Multiple chemical sensitivity (MCS) is a chronic clinical condition characterized by the appearance of a group of symptoms caused by exposure to chemical compounds that are tolerable for the general population [1,2]. The term, proposed by Cullen in 1987 [3], has the following characteristics:

- It is an acquired syndrome following documented environmental exposure that has objectively produced negative health effects.
- Symptoms affect multiple organs or systems and vary in magnitude depending on environmental stimuli.
- The symptoms are related to the levels of chemical agents directly, measurable levels and well below those considered toxicologically harmful.
- No evidence of organic damage is present.

The etiopathogenesis of MCS remains unknown; different possible origins of the disease have been described in the literature, such as psychiatric origins, but the cause is unclear [4]. It seems that central sensitization, an amplified response of the central nervous system to peripheral stimuli [5,6], may be an important factor contributing to the clinical manifestations of this disease [7]. Alterations in the metabolic activation of xenobiotic compounds have been established in people affected by MCS due to the presence of genetic polymorphisms. Specifically, there has been a significant relationship with the Superoxide Dismutase 2 (SOD2) [2]. Another study found that PON1 polymorphisms may play an additional role in patients with MCS who also have anxiety and depression [8].

MCS is a syndrome that affects mainly middle-aged women [4,9,10,11,12,13] with no differences related to educational, socioeconomic or ethnic background [14,15,16].

In some European countries, in the United States and in Japan, prevalence is estimated to be between 8 and 33% [11,17,18,19,20,21,22]. In Spain it has been recognized as a disease since 2014 and is coded under the heading 78.40 of the ICD 10 (International Classification of Diseases) [23]. We would like to point out that no prevalence study has yet been published in this country.

In order to make the diagnosis, it is necessary to meet the criteria established by consensus in 1999 [24] (Table 1). 

As a diagnostic method for this disease, in the United States (USA), Miller and Prihoda (1999) developed a self-administered global questionnaire, QEESI (Quick Environmental Exposure and Sensitivity Inventory), which has also been used in other countries such as Japan, Korea and Denmark [21,25,26,27,28,29,30]. In Spain, it has been translated and adapted cross-culturally and is used as a diagnostic tool [31] as it is considered a reliable, sensitive and specific instrument to measure the degree of affectation produced by MCS. Currently it is also used as a tool to assess the severity of symptoms and their impact on activities of daily living, to make the prognosis of the clinical picture and to identify triggering agents [10].

In Spain there are very few studies focused on the most frequent symptoms of MCS considering age groups and gender. These studies are necessary to be able to make a comparison with the situations described in other countries [1,32].

Numerous investigations have detected the need to broaden the study of these patients to promote a better understanding of the approach to this pathology [1,33,34,35,36,37]. It is known that an earlier diagnosis would favor a better evolution of the clinical picture and an improvement in the quality of life [38,39,40,41].

The main goals of this study were to describe the sociodemographic profile of our cohort of patients with MCS, to find the most frequent symptoms both at the onset of the disease and at the present time and to describe them by age group.

## 2. Materials and Methods

### 2.1. Design

An analytical observational case-control study was performed.

### 2.2. Scale 3 Questionnaire, QEESI—Initial Symptoms of MCS

The QEESI questionnaire is made up of 5 scales, each of which measures different aspects of the pathology valued from 1 to 10 according to the intensity of the affectation [10,31]. Scale 3: “Severity of symptoms” has been analyzed for this study.

A second questionnaire was included with questions related to the symptomatology presented by the patients at the onset of the disease MCS (Table 2).

### 2.3. Study Framework

The study was conducted in Spain, and data collection took place during the period from January 2020 to February 2021.

### 2.4. Definition of the Study Population

A case-control study was performed. The participants were women and men, over 18 years of age and of Spanish nationality.

Cases (criteria used to define a case):

The case group consisted of individuals with MSC who met the diagnostic criteria established by consensus (Table 1) [24].

Case recruitment was obtained through two associations of patients affected by MSC: Associations Network SFC–SQM (Síndrome de Fatiga Crónica–Sensibilidad Química Múltiple) (Chronic Fatigue Syndrome–Multiple Chemical Sensitivity) (https://www.sfcsqm.com/, accessed on 27 September 2022) and EQSDS (Electro y Químicos Sensibles por el Derecho a la Salud) (Electro and Sensitive Chemicals for the Right to Health) (https://electroyquimicosensibles.org/, accessed on 27 September 2022), both nationwide. The questionnaire QEESI [31] was distributed along with all the information on the study, the guarantee of confidentiality and the informed consent required to participate in the study.

Controls (criteria used to define control):

The control group consisted of individuals not diagnosed with MCS, recruited through social networks. The QEESI questionnaire [31] was distributed along with all the study information, the guarantee of confidentiality and the informed consent required to be able to participate in the study. The inclusion criteria for cases and controls are included in Table 3 below.

The total sample size was 70 patients in the case group and 140 in the control group, meaning the case/control ratio was 1/2, with the same sex and age.

Both the case group and the control group completed the same QEESI questionnaire format, Scale 3.

Subjects were stratified by sex (male–female) and age groups (31–40 years, 41–50 years, 51–60 years and ≥61 years).

### 2.5. Data Collection

Data collection was conducted using the Google Forms platform. A form was prepared having all the variables of the QEESI questionnaire, Scale 3, which was the object of the study. Each participant accessed the form after completing the informed consent form correctly.

The data collected were downloaded to an Excel file, guaranteeing the protection of personal data and the anonymity of the results.

### 2.6. Variables of Interest

The variables studied in the two study groups were as follows:

- Demographic variables: age and sex.
- QEESI questionnaire variables, Scale 3: severity of symptoms (Table 2). Each item was evaluated on a numerical scale from 1 to 10 according to severity, with 1 representing the least severity and 10 the most severity, as in the QEESI questionnaire scale [31]. The item scores were divided into four intervals from least to most severe (Table A1 and Table A2).
- Variables of questionnaire Initial symptoms of MCS: in the group of cases, the variables related to the symptoms suffered at the onset of the disease were also studied (Table 2).

### 2.7. Statistical Analysis

Statistical analysis was performed using the IBM SPSS software version 25 for Windows. Qualitative variables were described by frequencies and percentages, quantitative variables by medians and interquartile range (IQR Q1–Q3).

A bivariate analysis was conducted to assess the size of the association between the different variables studied, and the results were presented with the Odds Ratio (OR) and their corresponding 95% confidence intervals (95% CI). The “*p* value” considered statistically significant was *p* < 0.05.

### 2.8. Ethical Considerations

This study was approved by the Ethics Committee of the Rey Juan Carlos University of Madrid, Spain, registration number: 1706201910319. All subjects gave their consent before participating in the study, thus following the ethical principles of the Declaration of Helsinki [42]. All information obtained from the conduct of the present study was considered confidential in accordance with Organic Law 15/1999 of 13 December 1999 on the Protection of Personal Data [43] and the Organic Law 3/2018 of 5 December 2018 on the Protection of Personal Data and the Guarantee of Digital Rights [44].

## 3. Results

### 3.1. Description of the Sample Population

A total of 210 people took part in this study (70 cases and 140 controls), with a mean age of 51 years, with the following distribution by gender and age groups (Table 4).

The most notable characteristics found in the sample of cases were a greater predominance of women (*n* = 66, 94.3%), the difference in age groups in that the mean age for women, 50.67 years (±8.76), was notably lower than the mean age for men, 56.75 years (±12.12), and the higher frequency of the disease in women in the age group between 41 and 50 years (*n* = 29, 43.9%).

### 3.2. Symptoms

#### Initial Disease Profile

In this study, 41% (*n* = 25) of the sick women with MSC reported presenting at the onset of the disease with a single symptom, compared to 59% (*n* = 36) who reported polysymptomatology (Table 5).

The symptoms that appeared most often at the onset of the disease were those related to the airway and mucous membrane (*n* = 42, 68.9%), followed by gastrointestinal symptoms (*n* = 20, 32.8%). The least often occurring symptoms were those related to nervousness, irritability or depression (*n* = 2, 3.3%).

When analyzing the sample of women by age group, the following data were highlighted: the 31-to-40-years age group had the highest frequency of women with one symptom (*n* = 10, 50%), related to the airway and mucous membrane (*n* = 15.75%), followed by head-related symptoms (*n* = 8, 40%). In the 41-to-50-years age group, seven subjects (43.8%) suffered only one symptom, the most frequent being related to the airway and mucous membrane (*n* = 11, 68.8%), followed by head-related symptomatology and neuromuscular alterations with the same frequency (*n* = 4, 25%). It should be noted that no case presented symptoms related to the genitourinary system. In the 31-to-40-years age group, 50% of the patients (*n* = 10) presented polysymptomatology at the onset of the disease (Table 5).

The sample of men diagnosed with MCS was 5.7% (*n* = 4) of the total sample of cases. In them, the most frequent symptoms at the onset of the disease were those related to skeletal muscle disorders, airway and mucous membrane disorders and gastrointestinal symptoms, all in 50% (*n* = 2).

In the analysis of the sample of men by age groups, the following results stand out: the 41-to-50-years age group presented the highest frequency of individuals with only one symptom (*n* = 1, 100%), with these relating to the airway and mucous membrane alterations, and the number of individuals in the 21-to-30-years age group with one symptom was 100% (*n* = 1), related to skin alterations. Among those men who presented various symptoms at the onset of the disease, 100% were older than 51 years (Table 5).

### 3.3. Severity of the Symptom

**Figure 1:**
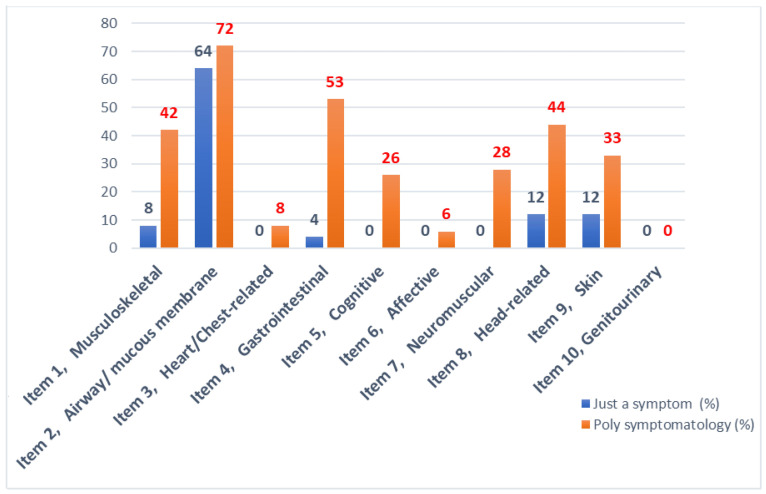
Percentage of patients with one symptom and with polysymptomatology at the onset of the disease and percentage of patients presenting the symptom today.

**Figure 2:**
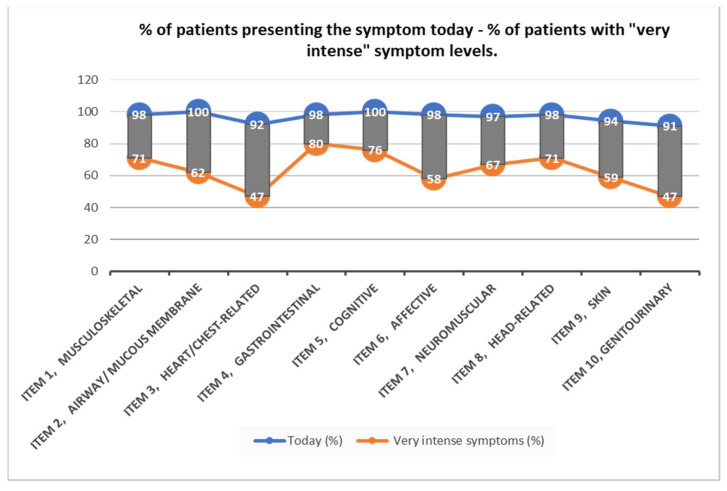
Percentage of patients presenting the symptom today and percentage of patients with “very intense” symptom levels.

The comparative analysis of the number of symptoms presented by women with MCS at the onset of the disease is represented in Figure 1. Among the symptoms presented by patients today, we detect a greater intensity of symptoms related to the digestive system (80%) and the neurological system (76%) followed by musculoskeletal disorders and headaches in the same proportion (71%) (Figure 2).

**Figure 3:**
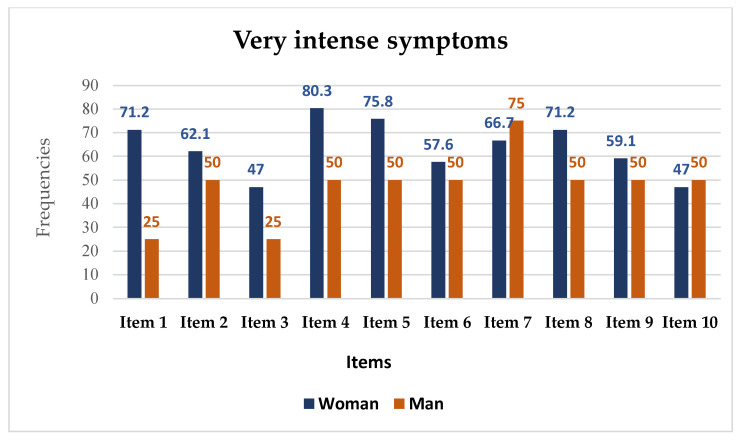
Frequency of very intense symptoms.

When comparing symptoms at the onset of the disease, looking at those with “very intense symptomatology” severity level, the vast majority are more frequent in women than in men, with the exception of symptoms related to the neuromuscular alterations and genitourinary symptoms, which are more frequent in men than in women (Figure 3), (see details in Appendix A—Table A1 and Table A2).

### 3.4. Bivariate Analysis

Regarding the study of symptoms, Table 6 shows the bivariate analysis of the symptoms in which the association with MCS disease has been found both by groups of women and by age groups. The symptoms at disease onset in women with MCS, which showed a statistically significant association (reported symptoms and MCS diagnosis), were cognitive alterations (OR = 31.25, 95% CI = 13.79–70.80), heart/chest problems (OR = 22.49, 95% CI = 8.14–62.13), neuromuscular problems (OR = 20.00, 95% CI = 9.13–43.78) and head-related symptomatology (OR = 19.29, 95% CI = 9.05–41.12). By age group, the following are observed: In the 31–40 years age group, only the symptom of cognitive alterations (OR = 32.50, 95% CI = 2.38–443.14) appears significantly; in the 41–50 years age group, the symptoms that stand out are related to heart/chest problems (OR = 53.20, 95% CI = 6.46–437.48) and cognitive alterations (OR = 33.22, 95% CI = 9.67–114.05). In the 51–60 age group, neuromuscular problems (OR = 76.00, 95% CI = 12.62–457.51) and head-related symptomatology (OR = 27.00, 95% CI = 6.35–114.66) were statistically significant. However, in the age group over 61 years, the most notable symptoms were those related to the musculoskeletal system (OR = 36.00, 95% CI = 4.28–302.80), affective disorders (OR = 28.50, 95% CI = 2.64–306.63) and gastrointestinal problems (OR = 21.00, 95% CI = 2.86–153.75) (Table 6). The sample referenced in the study does not offer significant data in relation to the bivariate analysis (OR) in men, probably due to the small number of men who participated.

## 4. Discussion

One of the most important problems when diagnosing MCS is the lack of a common pattern due to the variability of symptoms [3]. MCS today has no symptomatic pattern in relation to frequency, sex or age of onset. Previous studies such as those by Genuis SJ. [45], and Azuma et al. [21] have confirmed that a cessation of symptoms improves the prognosis of the disease. Due to the variability of the symptoms associated with the disease [46,47], it is necessary to take a multidisciplinary approach to the disease.

There are studies aimed at finding the symptoms of this disease and its relationship with different environmental factors and the person’s social and work environments, but very few studies have found epidemiological results on MCS, and none have related the symptoms of the disease by age group. The novelty reported in our study is data related to the frequency of symptomatology presentation according to age groups.

As main characteristics of the MCS patient profile, our study has reported a greater predominance of women affected with MCS (94%), as occurs in other studies conducted in different countries [4,9,10,11,12,13], with a mean age of 50.67 ± 8.76 years, which coincides with the study carried out by Alobid et al. [48].

In the bivariate analysis, no statistically significant results were found with respect to symptomatology in men with MCS. This result may be promoted by the small sample found; this event coincides with other studies conducted on MCS and found in the literature consulted [10,28].

In relation to the first symptoms manifested, although in the literature we have not found any study that typifies the first symptoms of the disease by age range, in our study 59% of the women manifested the appearance of more than one symptom at the onset of MCS. These symptoms are most often described after 11 years of age.

Thus, we find studies, such as the one by Andersson et al. [49], in which the presence of this disease is reported in children and adolescents.

In relation to symptomatology by age group, in our study the most frequent symptom declared in all age groups from 11 years onwards corresponds to item 2, burning or irritation in the eyes, breathing problems such as difficulty in breathing deeply, a lot of mucus and many respiratory infections (68.9%), being, in addition, the only item affected in 64% of the women who started the disease with a single symptom. This data could be justified because the main entry of chemicals into the body is through the respiratory and ocular routes. Although we have not found similar studies that typify these early symptoms, the work by Österberg et al. offers support for an irritant-based origin for MCS [50,51].

In the age groups 11 to 20 years and 21 to 30 years, another symptom that also appears often is the one corresponding to item 9, skin disorders, such as redness, rash, or dry skin with 37.5% and 45.5%, respectively. In the literature, we have found only one study carried out in adolescents where it inquiries about the presence of symptoms, mainly respiratory, without studying those related to the skin; it is the study carried out by Andersson et al. [49]. Our study shows that the onset of the disease in some cases occurs in childhood, in adolescence and also in those under 31 years of age, so it would be convenient to increase research in this population to see the symptomatologic behavior in younger people. The only found study was carried out by Pamela Reed Gibson [40], which placed the starting age at 32 years of age. Another finding present in the age group of 31-to-40-years relates to the symptoms corresponding to item 4, related to gastrointestinal disorders, and item 8, head-related symptomatology, with a percentage of 35% and 40%, respectively. These symptoms are described in more studies, but they do not specify the frequency according to age groups [1,9,22].

Our results show association in the following symptoms and age groups: in women of 31 to 40 years, a higher significance was obtained in item 5, difficulty to concentrate, remember things or make decisions (OR: 32.50, 2.38–443.14 *p* = 0.002); however, in the literature, the description of this symptom is reported in the MCS with significance, but without predominance in age group [10].

Other discovered results with significant association are the symptoms related to item 5, difficulty in concentrating, remembering things or making decisions (OR: 31.25, 13.79–70.80 *p* = 0.000); this symptom coincides with those reported in the studies by Vuokko et al. [34] and by Del Casale et al. [1]; second, the symptom corresponding to item 3 was found, problems in the heart or chest (tachycardia, arrhythmias, extrasystoles or pain) (OR: 22.49, 8.14–62.13 *p* = 0.000), although it appeared less frequently in the literature. However, its presence raises the need for further research in this area [52,53]. In women affected by MCS, the symptoms with association correspond to item 7, problems with balance or coordination of movements, numbness or tingling or difficulty in focusing vision (OR: 20.00, 9.13–43.78 *p* = 0.000); this symptom also was described in studies such as the one conducted by Nogué et al. and by Hojo et al. [10,32].

In the 51-to-60-years age bracket, symptoms such as problems with balance or coordination of movements, numbness, tingling or difficulty in focusing vision (OR 76.00 12.62–457.51 *p* = 0.000), headaches or sensation of pressure in the head (OR: 27.00 6.35–114.66 *p* = 0.000), and gastrointestinal disorders (OR 19.51 4.65–81.91 *p* = 0.000) stand out to a large extent. Previous studies [10,14] confirm that MCS affects mostly middle-aged women; in our sample, we observed a greater participation of women in the 41-to-50-years and 51-to-60-years age brackets, since these are the age intervals during which more association is reported.

Finally, in the age group ≥61 years, item 1, musculoskeletal problems (pain, cramps, contracture or weakness) (OR: 36.00 4.28–302.80 *p* = 0.000), and psychological disorders/affective (OR: 28.50 2.64–306.63 *p* = 0.001) were found This symptom is described in other studies such as the one conducted by Skovbjerg et al. where an association between MCS and psychological disorders was reported [54], but without specifying the age of the patients. In our study, symptoms related to the psychic sphere appear more frequently in women over 61 years of age; Storino V et al. argue that MCS is an underdiagnosed disease [46], as the lack of a common pattern and the lack of knowledge of the professionals themselves, leads to many years of suffering from the disease, with these symptoms appearing more predominantly in older women. It would be interesting to conduct other studies to assess whether menopause has a significant relationship with the aggravation of these symptoms in MCS.

In the case of men affected by MCS, no statistical significance was found, and although MCS is clearly a disease of higher prevalence in women, it is necessary to define its profile in men to achieve a better early diagnosis and better management of the disease.

When analyzing Scale 3 (severity of symptoms) of the QEESI questionnaire, our findings for women showed a higher frequency in the very intense symptomatology interval (8–10 points). No studies have been found that break down the scores of the Scale 3 of the QEESI questionnaire into intervals according to intensity. In relation to the age variable, there are few studies in which a segmentation by age group has been performed. These studies are those conducted by Hojo et al. [9] and Azuma et al. [21] in Japan and the study by Johansson et al. [55] in Sweden, although these studies analyzed different issues from those we present in our research. We affirm that studying the MCS population according to age bracket may supply more complete data considering this variable.

## 5. Strength and Limitation of the Study

The main strength of this study is to offer results of MCS symptomatology by groupings set up in age/gender ranges. Regarding the limitations of the study, one limitation is the small number of men who took part in the project, which can be justified by the clear predominance of pathology in women over men. Another limitation met was the difficulty of access to obtain the sample; it would be interesting in future research to access a greater number of possible patients through the participation of more associations of patients affected by MCS. It would be interesting in future research, starting from scale 1 (chemical intolerance), to know the possible causative agents of the symptoms described.

## 6. Conclusions

This study revealed a general profile of the MCS patient: a woman of 50.67 ± 8.76 years, with very intense difficulty in concentrating, heart or chest alterations, neuromuscular problems accompanied by a headache or a sensation of pressure in the face and a frequent manifestation of gastrointestinal disorders. Most often, the first symptoms are airway and mucous membrane problems. 

By age group, the profiles of MCS patients in this study meet the following characteristics: people 21–30 years of age who declare as the first symptoms those related to the airway and mucous membrane alterations, musculoskeletal disorders, gastrointestinal problems and skin alterations; between 31 to 40 and 41 to 50 years of age, the disease started with symptoms related to the airway and mucous membrane alterations, followed by head-related symptoms; in participants between 51 to 60 years of age, the symptoms were also those related to the airway and mucous membrane alterations.

A polysymptomatic onset of the disease occurs more often in women 21 to 30 years old, while in men it is seen among participants older than 51 years of age.

Based on the results obtained, our contribution on the most frequent pattern of symptoms reported by age group and sex will allow us to obtain an early diagnosis of the disease, trying to improve its prognosis and treatment.

It is a proposed that we conduct new research studies to be able to set up the validation of a symptomatic profile by age group and sex, which will contribute to further expanding the scientific knowledge available on MCS, and to be able to supply adequate social and health care coverage to patients affected by MCS.

## Figures and Tables

**Table 1:** Consensus criteria for the diagnosis of MCS.

Criteria for Diagnosis of MCS [24]	
1.	The symptoms are reproducible with (repeated chemical) exposure.
2.	The condition is chronic.
3.	Low levels of exposure (lower than previously or commonly tolerated) result in manifestations of the syndrome.
4.	The symptoms improve or resolve when the incitants are removed.
5.	Responses occur to multiple chemically unrelated substances.
6.	Symptoms involve multiple organ systems.

**Table 2:** Scale 3 QEESI questionnaire. Initial symptoms of MCS.

Scale 3, QEESI Questionnaire (Symptoms)	
**Item**	**Definition**
**Item 1**	Problems with your muscles or joint, such as pain, aching, cramping, stiffness or weakness
**Item 2**	Problems with burning or irritation of your eyes, or problems with your airway or breathing, such as feeling short of breath, coughing or having a lot of mucus, post-nasal drainage or respiratory infections
**Item 3**	Problems with your heart or chest, such as a fast or irregular heart rate, skipped beats, your heart pounding or chest discomfort
**Item 4**	Problems with your stomach or digestive tract, such as abdominal pain or cramping, abdominal swelling or bloating, nausea, diarrhea or constipation
**Item 5**	Problems with your ability to think, such as difficulty concentrating or remembering things, feeling spacey or having trouble making decisions
**Item 6**	Problems with your mood such as feeling tense or nervous, irritable, depressed, having spells of crying or rage or loss of motivation to do things that used to interest you
**Item 7**	Problems with balance or coordination, with numbness or tingling in your extremities or with focusing your eyes
**Item 8**	Problems with your head, such as headaches or a feeling of pressure or fullness in your face or head
**Item 9**	Problems with your skin, such as a rash, hives or dry skin
**Item 10**	Problems with your urinary tract or genitals, such as pelvic pain or frequent or urgent urination (For women: or discomfort or other problems with your menstrual period)
**Initial Symptoms of MCS**	
**Question**	**Definition**
**1**	At what age did the symptoms appear?
**2**	In what intensity?
**3**	What were the symptoms?

Legend. MCS: Multiple Chemical Sensitivity; QEESI: Quick Environmental Exposure and Sensitivity Inventory.

**Table 3:** Inclusion criteria to take part in the study.

Inclusion Criteria	
Cases	Controls
People of both sexes	People of both sexes
≥18 years	≥18 years
Patients with diagnosed MCS	Patients without diagnosed MCS
With explicit informed consent of the person	With explicit informed consent of the person

**Table 4:** Sociodemographic and clinical variables of cases and controls.

	Cases *n* = 70		Controls *n* = 140	
**Gender *n* (%) *n* (%)**				
** Females**	66 (94.3)		132 (94.3)	
** Males**	4 (5.7)		8 (5.7)	
**Median Age** **(Years) ± SD**	51.01 ± 8.98		51.03 ± 9.07	
**Age (years) grouped *n* (%) *n* (%)**				
31–40	7 (10.0)		14 (10.0)	
41–50	31 (44.3)		62 (44.3)	
51–60	20 (28.6)		40 (28.6)	
≥61	12 (17.1)		24 (17.1)	
	**Females *n* = 66**	**Males *n* = 4**	**Females *n* = 132**	**Males *n* = 8**
**Median Age (Years) ± SD**	50.67 ± 8.76	56.75 ± 12.12	50.64 ± 8.78	57.50 ± 11.89
**Age (years) grouped *n* (%) *n* (%)**				
** 31–40**	7 (10.6)	0	14 (10.6)	0
** 41–50**	29 (43.9)	2 (50.0)	58 (43.9)	4 (50.0)
** 51–60**	20 (30.3)	0	40 (30.3)	0
** ≥61**	10 (15.2)	2 (50.0)	20 (15.2)	4 (50.0)
**Scale 3, QEESI**				
**Median (Q1–Q3)**	77.0 (65.0–89.0)	65.5 (43.0–80.0)	27.0 (14.0–47.0)	15.0 (5.7–60.2)

Largest score of each scale 3, QEESI: 100 points.

**Table 5:** Frequency of cases, appearance of the first symptoms related to MCS by age groups and sex. Scale 3 of the QEESI questionnaire.

Woman	Total	0–10 Years	11–20 Years	21–30 Years	31–40 Years	41–50 Years	51–60 Years	≥61 Years
	*n* (%)	*n* (%)	*n* (%)	*n* (%)	*n* (%)	*n* (%)	*n* (%)	*n* (%)
	61 (100%)	2 (3.3%)	8 (13.1%)	11 (18.0%)	20 (32.8%)	16 (26.2%)	4 (6.6%)	0
**Item 1**	17 (27.9)	1 (50.0)	1 (12.5)	5 (45.5)	5 (25.0)	4 (25.0)	1 (25.0)	0
**Item 2**	42 (68.9)	0	5 (62.5)	8 (72.7)	15 (75.0)	11 (68.8)	3 (75.0)	0
**Item 3**	3 (4.9)	0	0	1 (9.1)	2 (10.0)	0	0	0
**Item 4**	20 (32.8)	1 (50.0)	2 (25.0)	5 (45.5)	7 (35.0)	3 (18.8)	2 (50.0)	
**Item 5**	9 (14.8)	0	1 (12.5)	4 (36.4)	1 (5.0)	3 (18.8)	0	0
**Item 6**	2 (3.3)	0	0	1 (9.1)	1 (5.0)	0	0	0
**Item 7**	10 (16.4)	0	1 (12.5)	2 (18.2)	3 (15.0)	4 (25.0)	0	0
**Item 8**	19 (31.1)	1 (50.0)	2 (25.0)	3 (27.3)	8 (40.0)	4 (25.0)	1 (25.0)	0
**Item 9**	15 (24.6)	1 (50.0)	3 (37.5)	5 (45.5)	3 (15.0)	3 (18.8)	0	0
**Item 10**	0	0	0	0	0	0	0	0
**Single symptom**	25 (41.0)	1 (50.0)	3 (37.5)	2(18.2)	10 (50.0)	7 (43.8)	2 (50.0)	0
**Polysymptomatic**	36 (59.0)	1 (50.0)	5 (62.5)	9 (81.8)	10 (50.0)	9 (56.2)	2 (50.0)	0
	**Total**	**0–10**	**11–20**	**21–30**	**31–40**	**41–50**	**51–60**	**≥** **61**
**Man**	*n* (%)	*n* (%)	*n* (%)	*n* (%)	*n* (%)	*n* (%)	*n* (%)	*n* (%)
	4 (100%)	0	0	1 (25%)	0	1 (25%)	1 (25%)	1 (25%)
**Item 1**	2 (50.0)	0	0	0	0	0	1 (100)	1 (100)
**Item 2**	2 (50.0)	0	0	0	0	1 (100)	1 (100)	0
**Item 3**	0	0	0	0	0	0	0	0
**Item 4**	2 (50.0)	0	0	0	0	0	1 (100)	1 (100)
**Item 5**	0	0	0	0	0	0	0	0
**Item 6**	0	0	0	0	0	0	0	0
**Item 7**	0	0	0	0	0	0	0	0
**Item 8**	0	0	0	0	0	0	0	0
**Item 9**	1 (25.0)	0	0	1 (100)	0	0	0	0
**Item 10**	0	0	0	0	0	0	0	0
**Single symptom**	25 (50.0)	0	0	1 (100)	0	1(100)	0	0
**Polysymptomatic**	2 (50.0)	0	0	0	0	0	1 (100)	1 (100)

**Table 6:** Odds Ratio and 95% CI of the symptoms in which association with MCS disease has been found in groups of women by age groups.

Women	Item	Symptom Scale 3	Cases 66 (%)	Controls 132 (%)	OR (CI 95%)	*p* Value
**Totals**	Item 1	Musculoskeletal	71.2%	15.9%	13.07 (6.44–26.54)	0.000
	Item 2	Airway/mucous membrane	62.1%	15.2%	9.18 (4.61–18.27)	0.000
	Item 3	Heart/Chest-related	47%	3.8%	22.49 (8.14–62.13)	0.000
	Item 4	Gastrointestinal	80.3%	18.2%	18.34 (8.66–38.86)	0.000
	Item 5	Cognitive	75.8%	9.1%	31.25 (13.79–70.80)	0.000
	Item 6	Affective	57.6%	15.9%	7.17 (3.65–14.09)	0.000
	Item 7	Neuromuscular	66.7%	9.1%	20.00 (9.13–43.78)	0.000
	Item 8	Head-related	71.2%	11.4%	19.29 (9.05–41.12)	0.000
	Item 9	Skin	59.1%	18.9%	6.18 (3.20–11.91)	0.000
	Item 10	Genitourinary	47%	8.3%	9.74 (4.44–21.33)	0.000
**31–40 years**	Item 5	Cognitive	71.40%	7.1%	32.50 (2.38–443.14)	0.002
**41–50 years**	Item 1	Musculoskeletal	69%	17.2%	10.66 (3.76–30.20)	0.000
	Item 2	Airway/mucous membrane	65.5%	13.8%	11.87 (4.07–34.59)	0.000
	Item 3	Heart/Chest-related	48.3%	1.7%	53.20 (6.46–437.48)	0.000
	Item 4	Gastrointestinal	82.80%	15.5%	26.13 (7.89–86.53)	0.000
	Item 5	Cognitive	79.3%	10.3%	33.22 (9.67–114.05)	0.000
	Item 7	Neuromuscular	65.5%	13.8%	11.87 (4.07–34.59)	0.000
	Item 8	Head-related	69%	13.8%	13.88 (4.69–41.07)	0.000
	Item 9	Skin	79.30%	24.1%	12.04 (4.08–35.51)	0.000
	Item 10	Genitourinary	55.2%	6.9%	16.61 (4.75–58.10)	0.000
**51–60 years**	Item 1	Musculoskeletal	75%	17.5%	14.14 (3.85–51.88)	0.000
	Item 2	Airway/mucous membrane	60%	12.5%	10.50 (2.87–38.35)	0.000
	Item 3	Heart/Chest-related	50%	5%	19.00 (3.57–100.96)	0.000
	Item 4	Gastrointestinal	85%	22.5%	19.51 (4.65–81.91)	0.000
	Item 5	Cognitive	70%	12.5%	16.33 (4.28–62.31)	0.000
	Item 6	Affective	60%	12.5%	10.50 (2.87–38.35)	0.000
	Item 7	Neuromuscular	80%	5%	76.00 (12.62–457.51)	0.000
	Item 8	Head-related	75%	10%	27.00 (6.35–114.66)	0.000
	Item 10	Genitourinary	45%	7.5%	10.09 (2.32–43.88)	0.001
**≥61 years**	Item 1	Musculoskeletal	80%	10%	36.00 (4.28–302.80)	0.000
	Item 4	Gastrointestinal	70%	10%	21.00 (2.86–153.75)	0.001
	Item 6	Affective	60%	5%	28.50 (2.64–306.63)	0.001

CI 95%: 95% confidence interval; Odds ratio: OR; Significance level *p* < 0.05; Scale 3 Symptoms (QEESI).

## Appendix A

**Table A1:** Frequencies of the scores assigned to each item of Scale 3 of the QEESI questionnaire in women.

		CASES					CONTROLS				
	Symptom Intensity	Woman	31–40	41–50	51–60	≥61	Woman	31–40	41–50	51–60	≥61
		*n* (%)	*n* (%)	*n* (%)	*n* (%)	*n* (%)	*n* (%)	*n* (%)	*n* (%)	*n* (%)	*n* (%)
		66 (100)	7 (10.6)	29 (43.9)	20 (30.3)	10 (15.2)	132 (100)	14 (10.6)	58 (43.9)	40 (30.3)	20 (15.2)
**Item 1**	No symptoms	1 (1.5)	-	-	1 (5.0)	-	24 (18.2)	3 (21.4)	10 (17.2)	7 (17.5)	4 (20.0)
	Mild symptomatology	3 (4.5)	1 (14.3)	2 (6.9)	-	-	38 (28.8)	3 (21.4)	19 (32.8)	8 (20.0)	8 (40.0)
	Moderate symptomatology	15 (22.7)	2 (28.6)	7 (24.1)	4 (20.0)	2 (20.0)	49 (37.1)	6 (42.9)	19 (32.8)	18 (45.0)	6 (30.0)
	Very intense symptoms	47 (71.2)	4 (57.1)	20 (69.0)	15 (75.0)	8 (80.0)	21 (15.9)	2 (14.3)	10 (17.2)	7 (17.5)	2 (10.0)
**Item 2**	No symptoms	-	-	-	-	-	32 (24.2)	3 (21.4)	9 (15.5)	13 (32.5)	7 (35.0)
	Mild symptomatology	6 (9.1)	1 (14.3)	2 (6.9)	1 (5.0)	2 (20.0)	45 (34.1)	5 (35.7)	25 (43.1)	12 (30.0)	3 (15.0)
	Moderate symptomatology	19 (28.8)	2 (28.6)	8 (27.6)	7 (35.0)	2 (20.0)	35 (26.5)	3 (21.4)	16 (27.6)	10 (25.0)	6 (30.0)
	Very intense symptoms	41 (62.1)	4 (57.1)	19 (65.5)	12 (60.0)	6 (60.0)	20 (15.2)	3 (21.4)	8 (13.8)	5 (12.5)	4 (20.0)
**Item 3**	No symptoms	5 (7.6)	-	2 (6.9)	2 (10.0)	1 (10.0)	64 (48.5)	5 (35.7)	32 (55.2)	18 (45.0)	9 (45.0)
	Mild symptomatology	11 (16.7)	2 (28.6)	5 (17.2)	3 (15.0)	1 (10.0)	39 (29.5)	6 (42.9)	17 (29.3)	12 (30.0)	4 (20.0)
	Moderate symptomatology	19 (28.8)	3 (42.9)	8 (27.6)	5 (25.0)	3 (30.0)	24 (18.2)	1 (7.1)	8 (13.8)	8 (20.0)	7 (35.0)
	Very intense symptoms	31 (47.0)	2 (28.6)	14 (48.3)	10 (50.0)	5 (50.0)	5 (3.8)	2 (14.3)	1 (1.7)	2 (5.0)	-
**Item 4**	No symptoms	1 (1.5)	-	1 (3.4)	-	-	33 (25.0)	2 (14.3)	18 (31.0)	8 (20.0)	5 (25.0)
	Mild symptomatology	4 (6.1)	-	1 (3.4)	2 (10.0)	1 (10.0)	43 (32.6)	4 (28.6)	19 (32.8)	12 (30.0)	8 (40.0)
	Moderate symptomatology	8 (12.1)	2 (28.6)	3 (10.3)	1 (5.0)	2 (20.0)	32 (24.2)	4 (28.6)	12 (20.7)	11 (27.5)	5 (25.0)
	Very intense symptoms	53 (80.3)	5 (71.4)	24 (82.8)	17 (85.0)	7 (70.0)	24 (18.2)	4 (28.6)	9 (15.5)	9 (22.5)	2 (10.0)
**Item 5**	No symptoms	-	-	-	-	-	40 (30.3)	3 (21.4)	19 (32.8)	9 (22.5)	9 (45.0)
	Mild symptomatology	3 (4.5)	-	1 (3.4)	1 (5.0)	1 (10.0)	44 (33.3)	6 (42.9)	20 (34.5)	14 (35.0)	4 (20.0)
	Moderate symptomatology	13 (19.7)	2 (28.6)	5 (17.2)	5 (25.0)	1 (10.0)	36 (27.3)	4 (28.6)	13 (22.4)	12 (30.0)	7 (35.0)
	Very intense symptoms	50 (75.8)	5 (71.4)	23 (79.3)	14 (70.0)	8 (80.0)	12 (9.1)	1 (7.1)	6 (10.3)	5 (12.5)	-
**Item 6**	No symptoms	1 (1.5)	-	1 (3.4)	-	-	41 (31.1)	5 (35.7)	19 (32.8)	10 (25.0)	7 (35.0)
	Mild symptomatology	5 (7.6)	2 (28.6)	-	1 (5.0)	2 (20.0)	42 (31.8)	4 (28.6)	20 (34.5)	12 (30.0)	6 (30.0)
	Moderate symptomatology	22 (33.3)	2 (28.6)	11 (37.9)	7 (35.0)	2 (20.0)	28 (21.2)	3 (21.4)	6 (10.3)	13 (32.5)	6 (30.0)
	Very intense symptoms	38 (57.6)	3 (42.9)	17 (58.6)	12 (60.0)	6 (60.0)	21 (15.9)	2 (14.3)	13 (22.4)	5 (12.5)	1 (5.0)
**Item 7**	No symptoms	2 (3.0)	1 (14.3)	-	1 (5.0)	-	50 (37.9)	5 (35.7)	20 (34.5)	16 (40.0)	9 (45.0)
	Mild symptomatology	3 (4.5)	1 (14.3)	1 (3.4)	-	1 (10.0)	42 (31.8)	5 (35.7)	20 (34.5)	13 (32.5)	4 (20.0)
	Moderate symptomatology	17 (25.8)	3 (42.9)	9 (31.0)	3 (15.0)	2 (20.0)	28 (21.2)	2 (14.3)	10 (7.6)	9 (6.8)	7 (35.0)
	Very intense symptoms	44 (66.7)	2 (28.6)	19 (65.5)	16 (80.0)	7 (70.0)	12 (9.1)	2 (14.3)	8 (13.8)	2 (5.0)	-
**Item 8**	No symptoms	1 (1.5)	-	-	1 (5.0)	-	34 (25.8)	2 (14.3)	15 (25.9)	13 (32.5)	4 (20.0)
	Mild symptomatology	3 (4.5)	1 (14.3)	1 (3.4)	1 (5.0)	-	51 (38.6)	6 (42.9)	23 (39.7)	13 (32.5)	9 (45.0)
	Moderate symptomatology	15 (22.7)	1 (14.3)	8 (27.6)	3 (15.0)	3 (30.0)	32 (24.2)	3 (21.4)	12 (20.7)	10 (25.0)	7 (35.0)
	Very intense symptoms	47 (71.2)	5 (71.4)	20 (69.0)	15 (75.0)	7 (70.0)	15 (11.4)	3 (21.4)	8 (13.8)	4 (10.0)	-
**Item 9**	No symptoms	4 (6.1)	1 (14.3)	-	2 (10.0)	1 (10.0)	27 (20.5)	2 (14.3)	11 (19.0)	6 (15.0)	8 (40.0)
	Mild symptomatology	6 (9.1)	2 (28.6)	1 (3.4)	2 (10.0)	1 (10.0)	44 (33.3)	5 (35.7)	22 (37.9)	11 (27.5)	6 (30.0)
	Moderate symptomatology	17 (25.8)	2 (28.6)	5 (17.2)	7 (35.0)	3 (30.0)	36 (27.3)	3 (21.4)	11 (19.0)	17 (42.5)	5 (25.0)
	Very intense symptoms	39 (59.1)	2 (28.6)	23 (79.3)	9 (45.0)	5 (50.0)	25 (18.9)	4 (28.6)	14 (24.1)	6 (15.0)	1 (5.0)
**Item 10**	No symptoms	6 (9.1)	-	1 (3.4)	3 (15.0)	2 (20.0)	55 (41.7)	5 (35.7)	26 (44.8)	15 (37.5)	9 (45.0)
	Mild symptomatology	10 (15.2)	-	4 (13.8)	3 (15.0)	3 (30.0)	40 (30.3)	3 (21.4)	17 (29.3)	14 (35.0)	6 (30.0)
	Moderate symptomatology	19 (28.8)	4 (57.1)	8 (27.6)	5 (25.0)	2 (20.0)	26 (19.7)	3 (21.4)	11 (19.0)	8 (20.0)	4 (20.0)
	Very intense symptoms	31 (47.0)	3 (42.9)	16 (55.2)	9 (45.0)	3 (30.0)	11 (8.3)	3 (21.4)	4 (6.9)	3 (7.5)	1 (5.0)

**Table A2:** Frequencies of the scores assigned to each item of Scale 3 of the QEESI questionnaire in men.

		CASES					CONTROLS				
	Symptom Intensity	Man	31–40	41–50	51–60	*>*61	Man	31–40	41–50	51–60	*>*61
		*n* (%)	*n* (%)	*n* (%)	*n* (%)	*n* (%)	*n* (%)	*n* (%)	*n* (%)	*n* (%)	*n* (%)
		4 (100)	-	2 (50.0)	-	n (%)	8 (100)	-	4 (50.0)	-	4 (50.0)
**Item 1**	No symptoms	-	-	-	-	-	3 (37.5)	-	2 (50.0)	-	1 (25.0)
	Mild symptomatology	2 (50.0)	-	1 (50.0)	-	1 (50.0)	3 (37.5)	-	2 (50.0)	-	1 (25.0)
	Moderate symptomatology	1 (25.0)	-	-	-	1 (50.0)	1 (12.5)	-	-	-	1 (25.0)
	Very intense symptoms	1 (25.0)	-	1 (50.0)	-	-	1 (12.5)	-	-	-	1 (25.0)
**Item 2**	No symptoms	-	-	-	-	-	3 (37.5)	-	2 (50.0)	-	1 (25.0)
	Mild symptomatology	-	-	-	-	-	2 (25.0)	-	1 (25.0)	-	1 (25.0)
	Moderate symptomatology	2 (50.0)	-	1 (50.0)	-	1 (50.0)	3 (37.5)	-	1 (25.0)	-	2 (50.0)
	Very intense symptoms	2 (50.0)	-	1 (50.0)	-	1 (50.0)	-	-	-	-	-
**Item 3**	No symptoms	-	-	-	-	-	5 (62.5)	-	3 (75.0)	-	2 (50.0)
	Mild symptomatology	-	-	-	-	-	1 (12.5)	-	1 (25.0)	-	-
	Moderate symptomatology	3 (75.0)	-	1 (50.0)	-	2 (100.0)	2 (25.0)	-	-	-	2 (50.0)
	Very intense symptoms	1 (25.0)	-	1 (50.0)	-	-	-	-	-	-	-
**Item 4**	No symptoms	-	-	-	-	-	2 (25.0)	-	1 (25.0)	-	1 (25.0)
	Mild symptomatology	-	-	-	-	-	3 (37.5)	-	2 (50.0)	-	1 (25.0)
	Moderate symptomatology	2 (50.0)	-	1 (50.0)	-	1 (50.0)	-	-	-	-	-
	Very intense symptoms	2 (50.0)	-	1 (50.0)	-	1 (50.0)	3 (37.5)	-	1 (25.0)	-	2 (50.0)
**Item 5**	No symptoms	-	-	-	-	-	2 (25.0)	-	1 (25.0)	-	1 (25.0)
	Mild symptomatology	1 (25.0)	-	-	-	1 (50.0)	2 (25.0)	-	1 (25.0)	-	1 (25.0)
	Moderate symptomatology	1 (25.0)	-	1 (50.0)	-	-	4 (50.0)	-	2 (50.0)	-	2 (50.0)
	Very intense symptoms	2 (50.0)	-	1 (50.0)	-	1 (50.0)	-	-	-	-	-
**Item 6**	No symptoms	-	-	-	-	-	3 (37.5)	-	2 (50.0)	-	1 (25.0)
	Mild symptomatology	2 (50.0)	-	1 (50.0)	-	1 (50.0)	2 (25.0)	-	1 (25.0)	-	1 (25.0)
	Moderate symptomatology	-	-	-	-	-	2 (25.0)	-	-	-	2 (50.0)
	Very intense symptoms	2 (50.0)	-	1(50.0)	-	1 (50.0)	1 (12.5)	-	1 (25.0)	-	-
**Item 7**	No symptoms	-	-	-	-	-	4 (50.0)	-	3 (75.0)	-	1 (25.0)
	Mild symptomatology	-	-	-	-	-	1 (12.5)	-	-	-	1 (25.0)
	Moderate symptomatology	1 (25.0)	-	1 (50.0)	-	-	2 (25.0)	-	-	-	2 (50.0)
	Very intense symptoms	3 (75.0)	-	1 (50.0)	-	2 (100.0)	1 (12.5)	-	1 (25.0)	-	-
**Item 8**	No symptoms	1 (25.0)	-	1 (50.0)	-	-	4 (50.0)	-	3 (75.0)	-	1 (25.0)
	Mild symptomatology	-	-	-	-	-	1 (12.5)	-	-	-	1 (25.0)
	Moderate symptomatology	1 (25.0)	-	1 (50.0)	-	-	1 (12.5)	-	-	-	1 (25.0)
	Very intense symptoms	2 (50.0)	-	-	-	2 (100.0)	2 (25.0)	-	1 (25.0)	-	1 (25.0)
**Item 9**	No symptoms	-	-	-	-	-	2 (25.0)	-	1 (25.0)	-	1 (25.0)
	Mild symptomatology	-	-	-	-	-	3 (37.5)	-	2 (50.0)	-	1 (25.0)
	Moderate symptomatology	2 (50.0)	-	1 (50.0)	-	1 (50.0)	3 (37.5)	-	1 (25.0)	-	2 (50.0)
	Very intense symptoms	2 (50.0)	-	1 (50.0)	-	1 (50.0)	-	-	-	-	-
**Item 10**	No symptoms	1(25.0)	-	1 (50.0)	-	-	4 (50.0)	-	3 (75.0)	-	1 (25.0)
	Mild symptomatology	1 (25.0)	-	-	-	1 (50.0)	-	-	-	-	-
	Moderate symptomatology	-	-	-	-	-	4 (50.0)	-	1 (25.0)	-	3 (75.0)
	Very intense symptoms	2 (50.0)	-	1 (50.0)	-	1 (50.0)	-	-	-	-	-

No symptoms: 0 points, Mild symptomatology: 1–3 points, Moderate symptoms: 4–7 points, very intense symptoms: 8–10 points.
